# Checkmate to CHK1 in T-cell ALL?

**DOI:** 10.18632/oncoscience.186

**Published:** 2015-08-12

**Authors:** Leonor M. Sarmento, João T. Barata

**Affiliations:** Instituto de Medicina Molecular, Faculdade de Medicina, Universidade de Lisboa, Lisbon, Portugal

**Keywords:** CHK1, replication stress, T-ALL, T-cell acute lymphoblastic leukemia, targeted therapy

DNA replication ensures accurate duplication of the original genetic information present in a cell in order for it to be properly transmitted to daughter cells. However, replication can be perturbed, for instance in rapidly dividing cancer cells, in a process referred to as replication stress (RS). Checkpoint kinase 1 (CHK1) is an essential component of the ATR-dependent DNA damage-response pathway that protect cells from RS by preventing replication fork collapse and activating homologous DNA repair. The ATR-CHK1 pathway is triggered upon exposure of single-stranded DNA that arises with the stalling of replication forks [1], and it is required to reset proper origin firing, and to promote fork stability and checkpoint activation, delaying mitosis until replication is completed and thereby avoiding mitotic catastrophe [2]. Whereas these functions point towards a tumor suppressor role for CHK1, mouse models modulating ATR-CHK1 expression and genetic evidence from human tumors suggest otherwise: *Atr* and *Chk1* knock-out models do not display higher tumor frequency; *Chk1* favors oncogene-induced transformation in mice; *CHK1* is frequently overexpressed in human cancers, while loss-of-function mutations are rare [2, 3]. Moreover, CHK1 affords protection against DNA damaging agents, a fact that prompted the use of CHK1 inhibitors as chemosensitizers [4]. Similarly, tumors whose oncogenic profile fuels RS were proposed to become addicted to ATR-CHK1 response [1, 2]. In our recent study published in Oncogene [5], we hypothesized that T-cell acute lymphoblastic leukemia (T-ALL), an aggressive hematological cancer arising from T-cell precursor clonal expansion, could be one of such tumors and showed that CHK1 plays a key role in T-ALL cell maintenance.

T-ALL cells tend to be highly proliferative due to a myriad of genetic lesions that culminate in cyclin-dependent kinase hyperactivation, and deregulated progression of S-phase that may impact on DNA replication [5, 6]. We found that T-ALL cells overexpressed CHK1 mRNA and protein as compared to normal hematopoietic progenitors. This was accompanied by aberrantly high CHK1 kinase activity, likely triggered by high basal levels of RS [5]. Experimental inactivation of CHK1, by a CHK1 selective inhibitor (PF-00477736) or by gene silencing, demonstrated that CHK1 is essential to control the accumulation of RS and to prevent apoptosis of T-ALL cells that appear to enter mitosis without having concluded DNA replication. Furthermore, accumulation of DNA damage in the context of CHK1 loss induced the activation of the ATM-CHK2 DNA double-strand break (DSB) response pathway, likely due to DSB formation upon the collapse of stalled replication forks. T-ALL apoptosis upon CHK1 inactivation was in the first instance dependent on ATM and caspase-3, since ATM inhibition prevented caspase-3 cleavage and rescued T-ALL cell viability despite sustained elevated amounts of RS markers [5].

Following the demonstration that T-ALL cells were eliminated using a CHK1 small molecule inhibitor as single agent, we showed that this effect was leukemia-specific, since normal T-cell progenitors were not sensitive to the low doses of PF-00477736 that killed primary T-ALL patient cells. Moreover, the *in vitro* anti-leukemia effect of PF-00477736 was not prevented by microenvironment pro-survival factors, and the potential clinical value of CHK1 inhibition was further demonstrated by the fact that PF-00477736 limited the growth of xenografted T-ALL tumors [5]. Interestingly, our preliminary analyses indicated that T-ALL cells expressing higher CHK1 levels appeared more sensitive to CHK1 pharmacological inhibition, suggesting that CHK1 expression could be a suitable drug response marker in T-ALL patients. As clinical trials against ATR-CHK1 pathway may be envisaged, this issue warrants extended T-ALL patient analysis.

T-ALL constitutes only a fraction of all ALL cases, but it associates with high-risk. Therapeutic options with less detrimental side-effects and/or effective upon relapse are most desired. Our findings defining CHK1 as a ‘subverted’ tumor suppressor that stands in T-ALL as a major guardian of leukemia cell survival, thereby formally acting as an oncogene, reinforce a new way of viewing the mechanisms of cancer progression [2] and may set the ground for anti-leukemia breakthrough approaches. In this context, it is important to understand the mechanisms of CHK1 upregulation in T-ALL. We thoroughly documented *CHK1* transcript overexpression in primary T-ALL [5]. However, how this occurs remains undetermined. Maybe transcription factors known to activate *CHK1*, such as E2F (downstream of G1/S-phase CDK activity) or MYC (downstream of NOTCH1), are involved in CHK1 overexpression in T-ALL. Or maybe as yet unidentified CHK1 regulatory elements are mutated or epigenetically altered. Curiously, in contrast to our findings, *Chk1* mRNA downregulation was documented in a murine T-ALL model [7]. A more integrative view of the role of CHK1 in T-ALL is therefore required. We believe CHK1 downregulation may occur at T-ALL initiation, driving genomic instability secondary to an increase in RS. As the pro-proliferative oncogenic program establishes and RS rises, leukemic cells are naturally selected for their ability to upregulate CHK1 as a means to maintain RS levels compatible with cell viability.
